# Clinical Experience With a New Type of Rhino-Larynx Electronic Endoscope PENTAX VNL-1530

**DOI:** 10.1155/DTE.1.57

**Published:** 1994

**Authors:** Masahiro Kawaida, Hiroyuki Fukuda, Naoyuki Kohno

**Affiliations:** 1 Department of Otolaryngology Tokyo Metropolitan Ohtsuka Hospital Tokyo, Japan 8-1, 2-chome Minamiohtsuka Toshima-ku Tokyo 170 Japan; 2 Department of Otolaryngology Keio University School of Medicine Tokyo, Japan 35 Shinanomachi Shinjuku-ku Tokyo 160 Japan; 3 Department of Otolaryngology Juntendo University School of Medicine Tokyo, Japan 1-1, 2-chome Hongo Bunkyo-ku Tokyo 113 Japan

## Abstract

We observed recordings of pictures obtained from patients with diseases of the larynx by using a new type of rhino-larynx electronic endoscope, PENTAXVNL-1530 connected to a video processor, PENTAX EPM-3300 (Asahi Optical Co., Ltd.). The electronic endoscope differs from the fiberoptic endoscope in that it contains a small light-sensitive charge coupled device (CCD) chip that is attached to the tip of the endoscope. This electronic endoscope has the smallest CCD camera of 5.1 mm in diameter, in the tip portion, and can be passed through the nasal passage into the laryngeal cavity. The dynamic image provided by this system is superior to that obtained by a flexible laryngofiberscope in resolution of the detail.

The system with this electronic endoscope was introduced and some clinical cases were presented.


*Diagnostic and Therapeutic Endoscopy,* Vol. 1, pp. 57–62
Reprints available directly from the publisher
Photocopying permitted by license only

© 1994 Harwood Academic Publishers GmbH
Printed in Malaysia

### Clinical Experience with a New Type of Rhino-larynx Electronic Endoscope PENTAX VNL-1530

MASAHIRO KAWAIDA*, HIROYUKI FUKUDA** and NAOYUKI KOHNO***

**Department of Otolaryngology, Tokyo Metropolitan Ohtsuka Hospital, Tokyo, Japan 8-1, 2-chome Minamiohtsuka, Toshima-ku, Tokyo 170, Japan*
***Department of Otolaryngology, Keio University School of Medicine, Tokyo, Japan 35 Shinanomachi, Shinjuku-ku, Tokyo 160, Japan*
****Department of Otolaryngology, Juntendo University School of Medicine, Tokyo, Japan 1-1, 2-chome Hongo, Bunkyo-ku, Tokyo 113, Japan*

*(Received October 7, 1993; in final form December 2, 1993)*

We observed recordings of pictures obtained from patients with diseases of the larynx by using a new type of rhino-larynx electronic endoscope, PENTAX VNL-1530 connected to a video processor, PENTAX EPM-3300 (Asahi Optical Co., Ltd.). The electronic endoscope differs from the fiberoptic endoscope in that it contains a small light-sensitive charge coupled device (CCD) chip that is attached to the tip of the endoscope. This electronic endoscope has the smallest CCD camera of 5.1 mm in diameter, in the tip portion, and can be passed through the nasal passage into the laryngeal cavity. The dynamic image provided by this system is superior to that obtained by a flexible laryngofiberscope in resolution of the detail.

The system with this electronic endoscope was introduced and some clinical cases were presented.

**KEY WORDS:** electronic endoscope, laryngoendoscope, flexible laryngofiberscope, diseases of the larynx, RGB sequencing system

### INTRODUCTION

It is important to correctly observe the larynx in patients with laryngeal diseases. Precise diagnosis of disease at an early stage leads directly to the proper treatment of the disease. Due to advances in electronic equipment, a rhino-larynx electronic endoscope has been developed with a small CCD (charge coupled device) camera in the tip portion, which is about 5.0 mm in diameter. The purpose of this paper is to report on a new type of rhino-larynx electronic endoscope system which allows for smooth insertion through the nasal passage into the laryngeal cavity.

### INSTRUMENTS AND METHODS

Electronic endoscopic examinations have been carried out using a new type of rhino-larynx electronic endoscope, PENTAX VNL-1530 (Asahi Optical Co., Ltd.) (Fig. 1). The dimensions and characteristics of this endoscope are shown in Table 1.

The electronic endoscope contains a small light-sensitive CCD chip that is attached to the tip of the endoscope as a miniature TV camera. The image detected by the CCD chip is passed in the form of an electronic signal, through the endoscope to a video processor, PENTAX EPM-3300 (Asahi Optical Co., Ltd.), which changes the signal to a form capable of being visualized on the TV monitor by the RGB sequencing system. Light supplied from a source in the video processor is passed through the light guide of the endoscope via a glassfiber bundle for illumination. It is possible to add various recording devices such as a videotape recorder, a videoprinter, and an automatic monitor photo unit to this system. A freeze-frame facility is also provided. A subscreen mode permits simultaneous viewing of frozen and moving images via the main screen and sub-screen. The other features of the endoscope, such as the control mechanisms of the tip, resemble those of conventional flexible laryngofiberscopes.

Address for correspondence: Dr. Masahiro Kawaida, Department of Otolaryngology, Tokyo Metropolitan Ohtsuka Hospital, 8-1, 2-chome Minamiohtsuka, Toshima-ku, Tokyo 170, Japan.

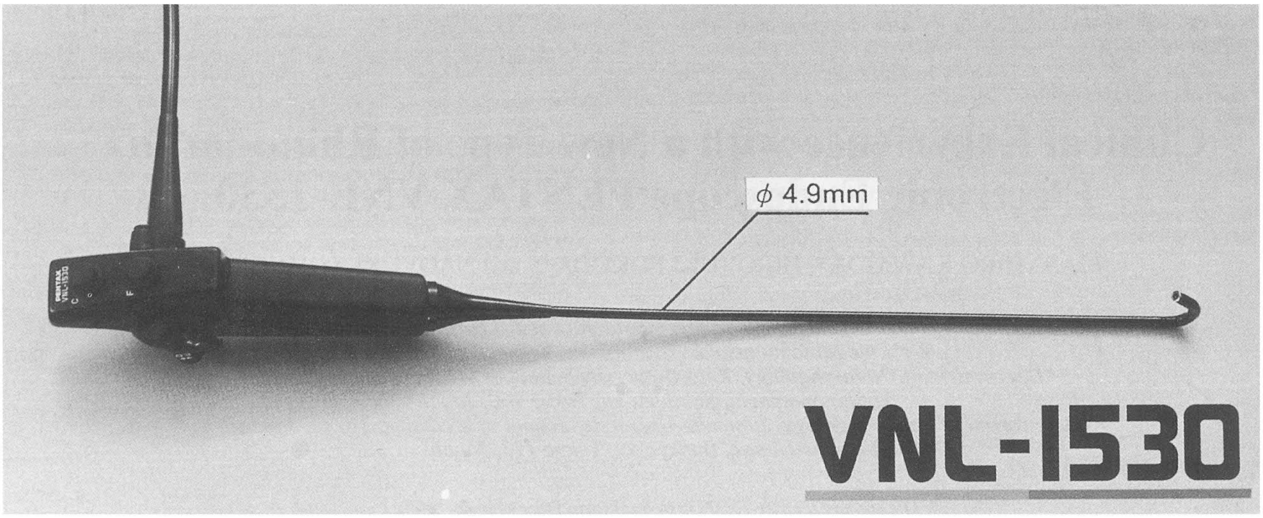

**Figure 1** Rhino-larynx electronic endoscope PENTAX VNL-1530 (Asahi Optical Co., Ltd.).

**Table 1** Rhino-larynx electronic endoscope PENTAX VNL-1530 (Asahi Optical Co., Ltd.): Dimensions and characteristics

| | | |
|---|---|---|
| *Optical system* | *Field of view* | *85°* |
| | *Direction of view* | *Forward viewing* |
| | *Depth of field* | *3–50 mm* |
| Bending section | Range of tip bending | Up 130°, Down 130° |
| Distal end | Outer diameter | 5.1 mm |
| Insertion tube | Outer diameter | 4.9 mm |
| Working length | | 300 mm |
| Total length | | 515 mm |
| Video processer | | PENTAX EPM-3300 (RGB seq. system) |

When this electronic endoscope is used in a patient, surface anesthesia with 4% lidocaine-HC*l* spray applied into the nasal cavity is performed. The patient is examined in the seated position. The endoscope is then passed through the nasal passage and introduced into the laryngeal cavity (Fig. 2).

### PATIENTS

Two patients were examined using this electronic endoscope system. Patient 1 was a 35-year-old woman who had vocal fold nodules. Patient 2 was a 50-year-old man who had a left vocal fold polyp.

### RESULTS

A picture taken during the examination is shown in Figure 2. Recorded pictures obtained by freeze-frame are shown in Figures 3a and 4a. In addition, pictures obtained by videography with a conventional flexible laryngofiberscope are also shown in Figures 3b and 4b. Figure 3 shows vocal fold nodules (patient 1). Figure 4 shows the left vocal fold polyp (patient 2).

The quality of the images obtained by the electronic endoscope system was outstanding. Color reproduction of the laryngeal mucosa appeared normal, and resolution was excellent.

The maneuverability of this rhino-larynx electronic endoscope is identical to that of conventional flexible laryngofiberscopes.

### DISCUSSION

Laryngoendoscopy began with the description of the first flexible laryngofiberscope in 1968 (Swashima and Hirose, 1968). Not long afterwards, the rigid laryngotelescope (Honda *et al.*, 1977) and curved laryngotelescope (Saito *et al.*, 1984) were developed as a rigid type of laryngoendoscope. More recently, video laryngoscopy using a laryngoendoscope connected to a color video camera and a videotape recording system was developed (Yoshida *et al.*, 1979; Yanagisawa *et al.*, 1981).

The flexible laryngofiberscope has the advantage that it can reach objects more easily. However, it has the disadvantage that the obtained image is very rough. The mechanical characteristics of the electronic endoscope, PENTAX VNL-1530 are identical to those of the flexible laryngofiberscopes. However, the electronic endoscope differs from the fiberoptic endoscope in that it contains a small light-sensitive CCD chip that is attached to the tip of the endoscope (Classen and Phillip, 1984; Matek *et al.*, 1984). This endoscope provides high quality, optimum brightness, superior color balance, and high resolution of the TV image to enable optimal diagnosis.

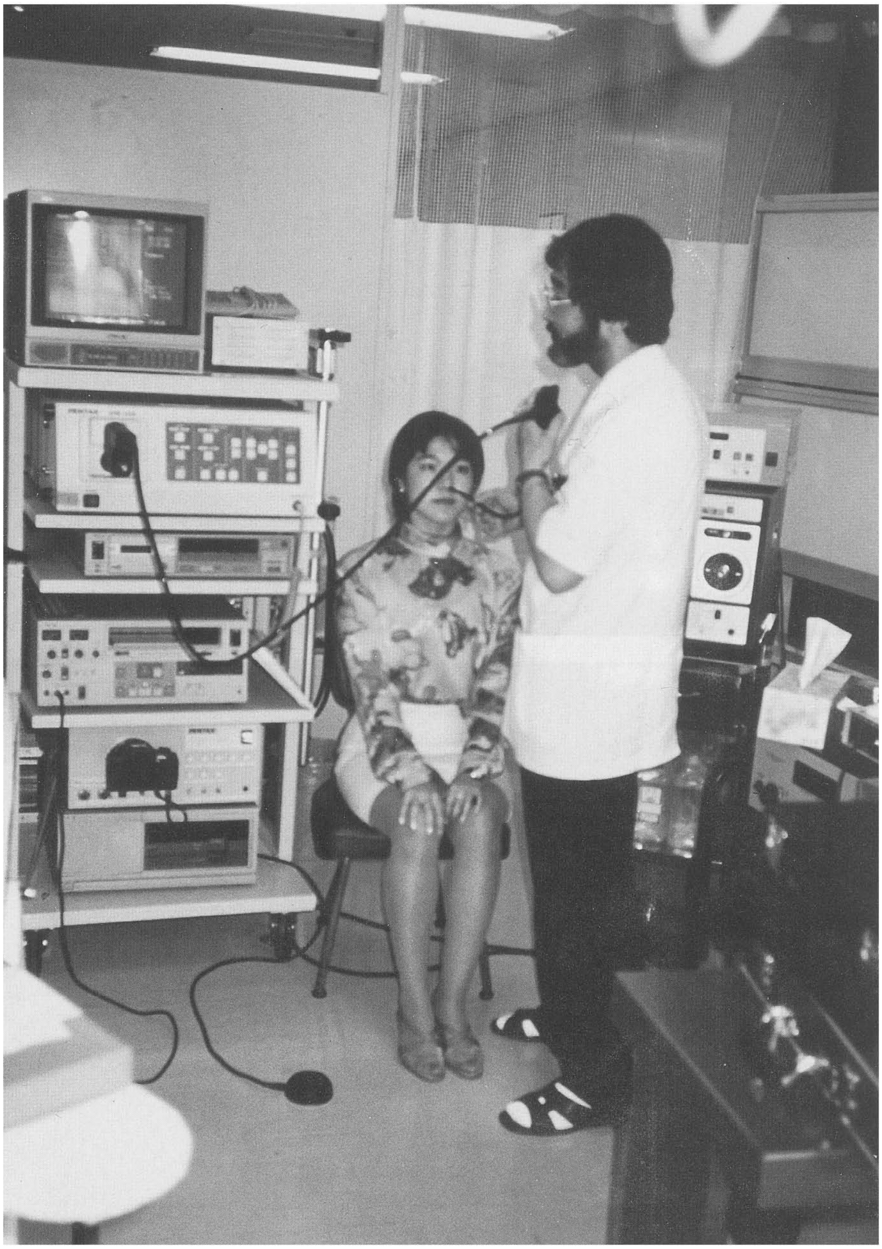

**Figure 2** The device in clinical use. The patient was examined in a sitting position and the endoscope was passed through the nasal passage.

Documentation of the entire examination on videotape is possible. Individual views can be photographed directly from the monitor screen using the freeze-frame facility and automatic monitor photo unit. Furthermore, it will also be possible using new techniques such as still video floppy discs to record the findings in a rapid manner.

The electronic endoscope, PENTAX VNL-1530 has a small CCD camera in its tip, which is 5.1 mm in diameter. Examination with this electronic endoscope system is easily performed through the nasal passage into the laryngeal cavity after only surface anesthesia. Patients experience less pain. The dynamic image provided by this system is superior to that obtained by conventional flexible laryngofiberscopes with regard to resolution of details. The main advantage is in the high quality video image reproduction on a TV monitor. This may help in education and better de-

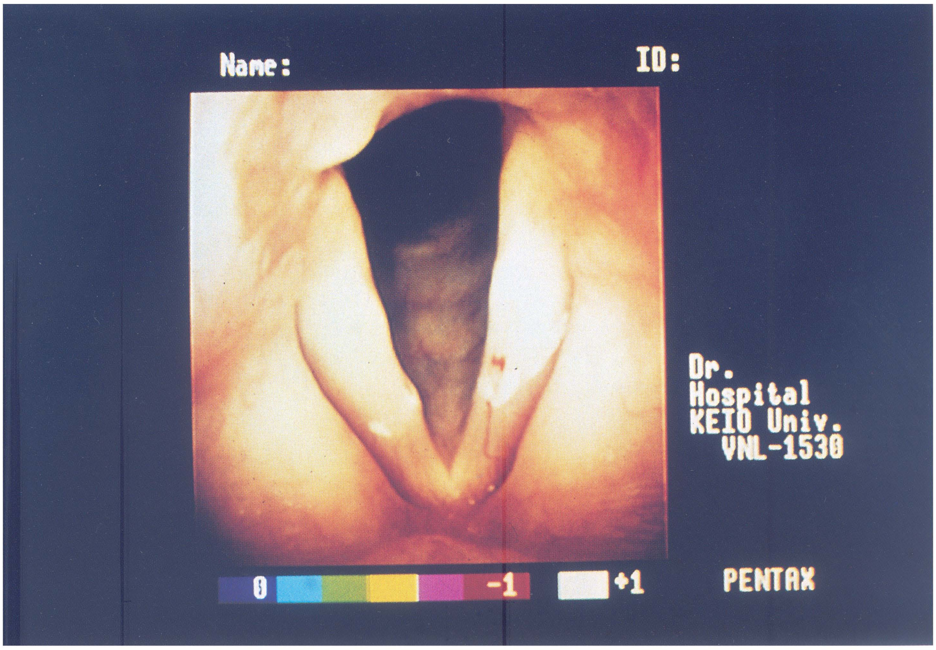

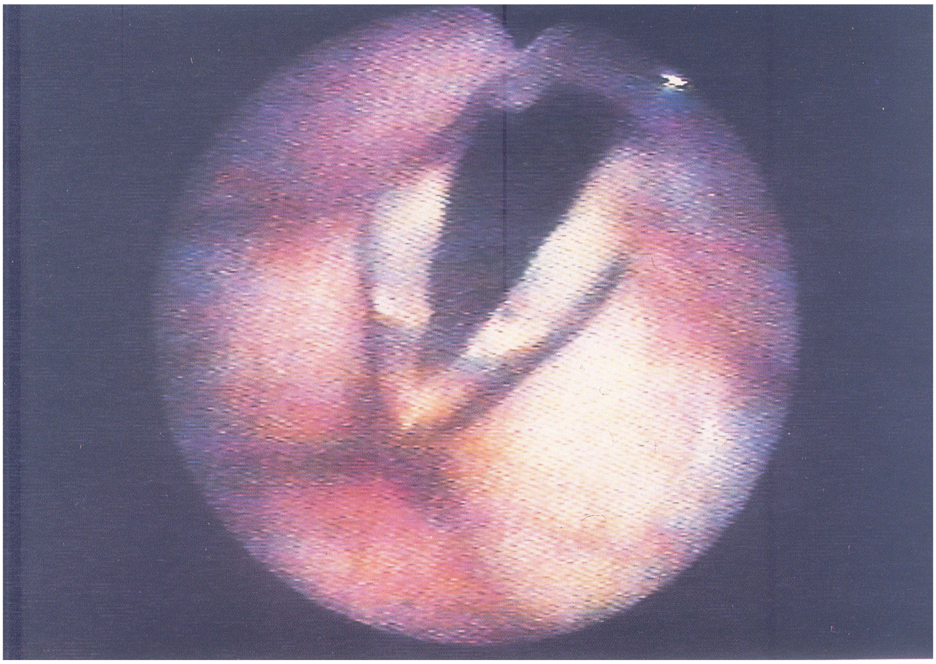

**Figure 3** Clinical findings of vocal fold nodules (patient 1). a, A picture photographed with the freeze-frame facility of this electronic endoscope system and automatic monitor photo unit. b, A picture obtained by videography with a conventional flexible laryngofiberscope.

finition of the image that the physician uses for diagnostic purposes. Through the use of the processor terminals, it is also possible to access various computer products.

There is one problem that must be addressed. Stroboscopic examination is the most practical way to determine the vibratory mode of the vocal folds during phonation in the field of laryngology (Yoshida *et al.*, 1979; Saito *et al.*, 1978; Fukuda *et al.*, 1990). However, it is difficult to keep a record of stroboscopic images using an electronic endoscope employing the RGB sequencing sys-

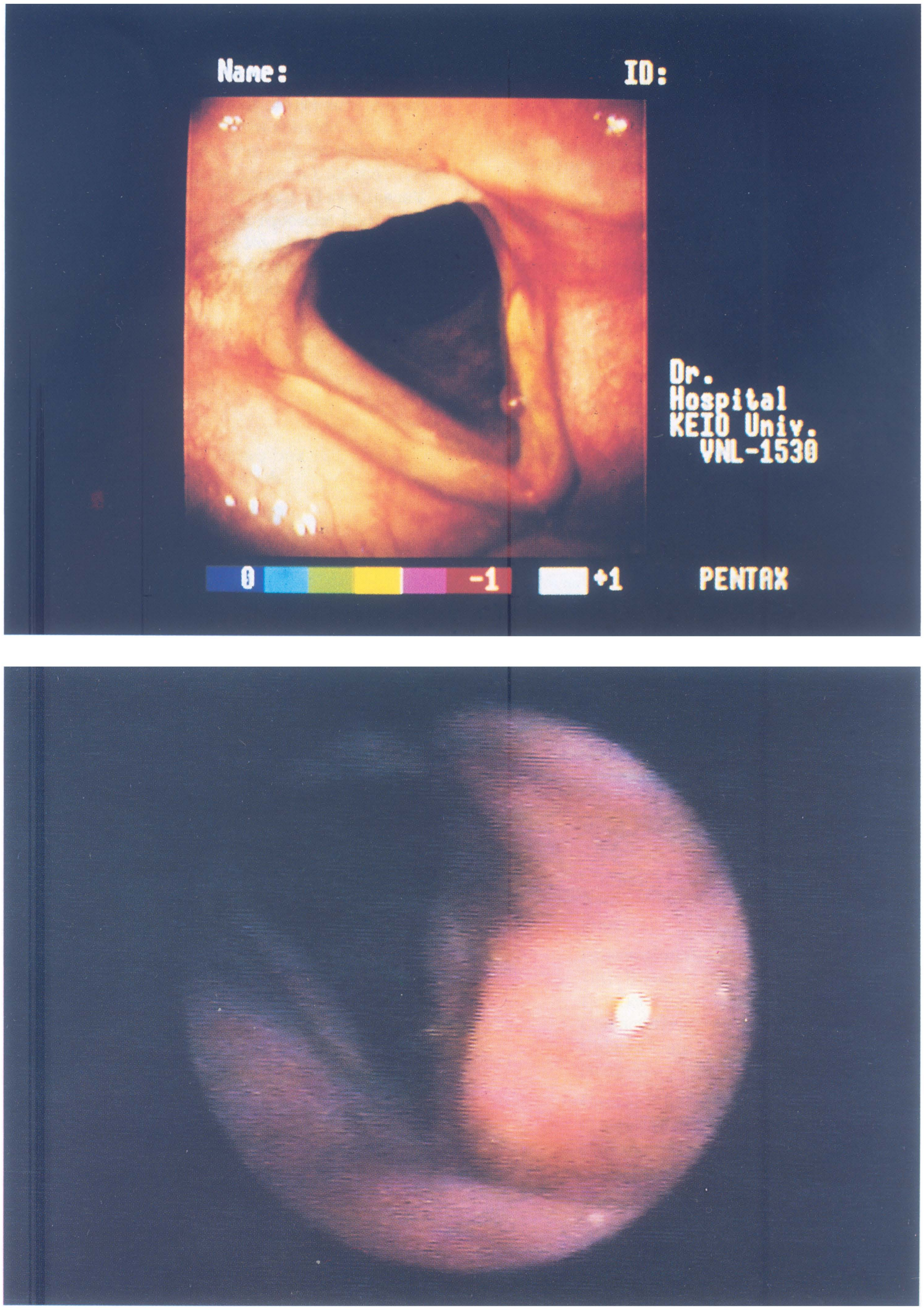

**Figure 4** Clinical findings of left vocal fold polyp (Patient 2). a: A picture photographed with the freeze-frame facility of this electronic endoscope system and automatic monitor photo unit. b: A picture obtained by videography with a conventional flexible laryngofiberscope.

tem. In order to observe and record stroboscopic images using an electronic endoscope, it will be necessary to develop a new type of rhino-larynx electronic endoscope containing a small color chip CCD system in the tip portion of about 5.0 mm in diameter.

### CONCLUSION

A newly developed rhino-larynx electronic endoscope that can be introduced through the nasal passage into the laryngeal cavity to observe and record the findings of the larynx is described. This electronic endoscope system provides high quality and high resolution of the TV image.

### REFERENCES

Classen M., Phillip J.: Electronic endoscopy of the gastrointestinal tract; Initial experience with a new type of endoscope that has no fiberoptic bundle for imaging. *Endoscopy*, 1984;16:16–19
Fukuda H., Kawaida M, Oki K. *et al*: Laryngostrobovideography using a flexible laryngofiberscope performed in conjunction with the phonatory examination. *Keio J Med*, 1990;39:102–105
Honda K., Kobayashi T., Tsubaki Y. *et al*: Observation and recording of laryngeal view by use of a modified oblique-angled type telescope. *J Jpn Bronchoesophagol Soc*, 1977;28:18–19
Matek W., Lux G., Riemann J. F., Demling L.: Initial experience with the electronic endoscope. *Endoscopy*, 1984;16:20–21
Saito S., Fukuda H., Kitahara S. *et al*: Curved laryngotelescope. *Laryngoscope*, 1984;94:1103–1105
Saito S., Fukuda H., Kitahara S. *et al*: Stroboscopic observation of vocal fold vibration with fiberoptics. *Folia Phoniatr*, 1978;30:241–244
Sawashima M., Hirose H.: New laryngoscopic technique by use of fiber optics. *J Acoust Soc Am*, 1968;43:168–169
Yanagisawa E., Casuccio J. R., Suzuki M.: Video laryngoscopy using a rigid telescope and video home system color camera. *Ann Otol Rhinol Laryngol.*, 1981;90:346–350
Yoshida Y., Hirano M., Nakajima T.: A video-tape recording system for laryngo-stroboscopy. *J Jpn Bronchoesophagol Soc*, 1979;30:1–5

